# Segmentation of incident lacunes during the course of ischemic cerebral small vessel diseases

**DOI:** 10.3389/fneur.2023.1113644

**Published:** 2023-03-23

**Authors:** Jessica Lebenberg, Ruiting Zhang, Lina Grosset, Jean Pierre Guichard, Fanny Fernandes, Eric Jouvent, Hugues Chabriat

**Affiliations:** ^1^APHP, Lariboisière Hospital, Translational Neurovascular Centre, FHU Neurovasc, Université Paris Cité, Paris, France; ^2^U1141, Université Paris Cité, Inserm, Neurodiderot, Paris, France; ^3^Department of Radiology, School of Medicine, The Second Affiliated Hospital of Zhejiang University, Hangzhou, China; ^4^APHP, Lariboisière Hospital, Department of Neurology, FHU NeuroVasc, Université Paris Cité, Paris, France; ^5^Faculté de Santé, Université Paris Cité, Paris, France; ^6^APHP, Lariboisière Hospital, Department of Neuroradiology, Université Paris Cité, Paris, France

**Keywords:** incident lacunes, CADASIL, cerebral small vessel disease, follow-up, clinical trial

## Abstract

**Background:**

Lacunes represent key imaging markers of cerebral small vessel diseases (cSVDs). During their progression, incident lacunes are related to stroke manifestations and contribute to progressive cognitive and/or motor decline. Assessing new lesions has become crucial but remains time-consuming and error-prone, even for an expert. We, thus, sought to develop and validate an automatic segmentation method of incident lacunes in CADASIL caused by cysteine mutation in the EGFr domains of the NOTCH3 gene, a severe and progressive monogenic form of cSVD.

**Methods:**

Incident lacunes were identified based on difference maps of 3D T1-weighted MRIs obtained at the baseline and 2 years later. These maps were thresholded using clustering analysis and compared with results obtained by expert visual analysis, which is considered the gold standard approach.

**Results:**

The median number of lacunes at the baseline in 30 randomly selected patients was 7 (IQR = [2, 11]). The median number of incident lacunes was 2 (IQR = [0, 3]) using the automatic method (mean time-processing: 25 s/patient) and 0.5 (IQR = [0, 2]) using the standard visual approach (mean time-processing: 8 min/patient). The complementary analysis of segmentation results is enabled to quickly remove false positives detected in specific locations and to identify true incident lesions not previously detected by the standard analysis (2 min/case). A combined approach based on automatic segmentation of incident lacunes followed by quick corrections of false positives allowed to reach high individual sensitivity (median at 0.66, IQR = [0.21, 1.00]) and global specificity scores (0.80).

**Conclusion:**

The automatic segmentation of incident lacunes followed by quick corrections of false positives appears promising for properly and rapidly quantifying incident lacunes in large cohorts of cSVDs.

## 1. Introduction

Cerebral small vessel diseases (cSVDs) represent a major cause of stroke (1). They are also the main source of vascular cognitive impairment and the second most common cause of dementia (2, 3). Moreover, cSVDs are responsible for covert brain lesions frequently detected on magnetic resonance imaging (MRI) in the general population, the prevalence of which increases considerably with aging (4). Their severity appears strongly related to aging, genetic variants, and exposure to vascular risk factors, particularly hypertension (5, 6). During the last decades, monogenic forms of cSVDs have been identified among the most progressive and severe types of such conditions. Although rare, these disorders are considered today as unique models to better understand the mechanisms underlying the progression of hereditary and sporadic cSVDs (7, 8).

Among the hallmarks of cSVDs are lacunes, detected using MRI, as small cavities of cerebrospinal fluid (CSF) appearing in the brain during their progression. Lacunes develop in the cerebral tissue after previous focal ischemic lesions, and more rarely after small hemorrhages. During the course of cSVDs, accumulating evidence shows that incident lacunes can also occur in the total absence of stroke manifestations while contributing to cognitive and/or motor decline (9). Incident lacunes also promote the development of cerebral atrophy and cortical thinning (10). Thus, assessing the number and the location of incident lacunes has become crucial for longitudinal studies on cSVD and a key outcome for clinical trials.

Quantitative studies of incident lacunes, even those derived from segmentation techniques, remain however largely based on the visual assessment of imaging data (11, 12). The related tasks, which are operator-dependent and time-consuming, might be difficult to apply to a large bundle of MRI exams. Moreover, these tasks appear particularly difficult in the presence of severely damaged brain tissue or the presence of multiple lesions mimicking lacunes, such as perivascular spaces (13). To overcome these difficulties as well as potential random operator errors, we aimed to improve the automation of the segmentation method to facilitate both the detection (number and location) and delineation of incident lacunes in cSVDs based on clinical MRIs. Herein, we present the results obtained with such an approach for measuring incident lacunes in CADASIL, a severe and progressive cSVD of hereditary origin.

## 2. Materials and methods

### 2.1. Patients

All patients included in this study had a genetically confirmed diagnosis of CADASIL caused by cysteine mutation in the EGFr domains of the NOTCH3 gene. They were followed by the National French Referral Center for Rare Vascular Diseases of the Eyes and Brain (CERVCO, https://www.cervco.fr). Every 18–24 months, they were systematically evaluated with standardized MRI including millimetric resolution 3D-T1 images. The study was approved by an independent ethics committee (updated agreement CEEI-IRB-17/388) and was conducted in accordance with the Declaration of Helsinki and guidelines for Good Clinical Practice and General Data Protection Regulation (GDPR) in Europe.

### 2.2. Dataset

Thirty patients were randomly selected for the present study. They were on average 53.7 years old [range: (32.3, 74.6)], and 57% were women. The baseline and the first follow-up 3D T1-weighted MRI (MPRAGE, GRAPPA acceleration factor 3, and sagittal acquisition) were acquired at 3T on a SIEMENS Skyra system, without denoising, with a ratio of the field of view dimension in the phase direction to the field of view dimension in the frequency direction at 100% and an interpolated resolution. A total of 10 out of 30 baseline scans were acquired with the following parameters for MRI: TR/TE/TI 1800/2.4/907 ms, voxel size of 0.4 mm × 0.4 mm × 0.9 mm, acquisition matrix of 320x320, field of view of 269 mm × 26 mm, and acquisition time of 3:59 min. The remaining baseline and all follow-up data were acquired with the following parameters: TR/TE/TI 2000/3.2/900 ms, voxel size of 0.4 mm × 0.4 mm × 0.4 mm, acquisition matrix of 288 × 288, field of view of 242 mm × 242 mm, and acquisition time of 4:01 min.

### 2.3. Segmentation of lacunes

**Figure 1:**
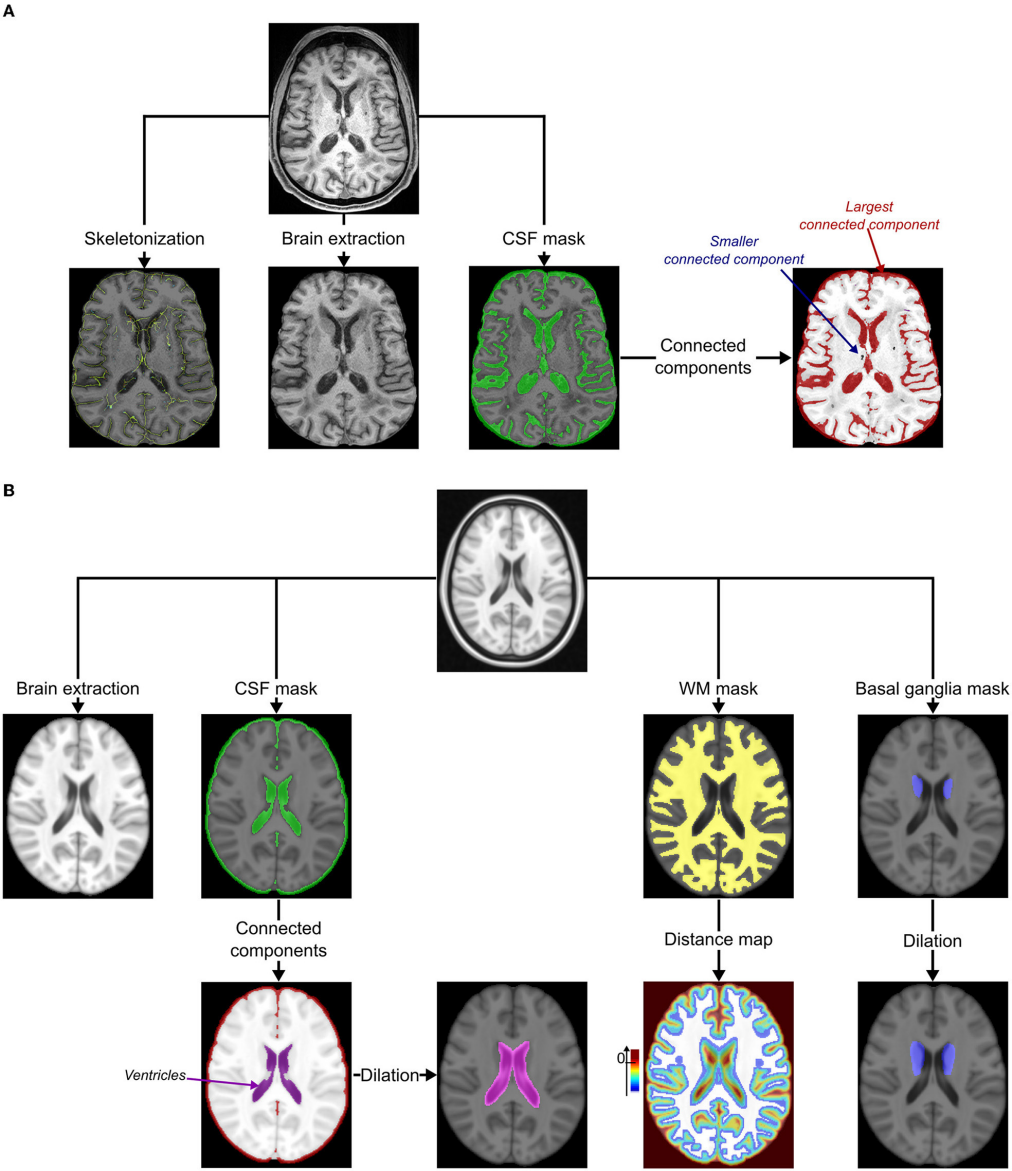
Pre-processing. **(A)** Pre-processing of MRIs under study. The cortical sulci were skeletonized using topological and regularization constraints applied to the excrescence of the union of the cerebrospinal fluid (CSF) and the gray matter in the brain (15). The bias of MRI was corrected using the N4 correction procedure (16) and both the brain and CSF masks were extracted using an iterative process based on Bayesian segmentation techniques (17). The connected components of the CSF mask were identified using the ANTs image processing tools (http://stnava.github.io/ANTs/): the largest represented the external CSF (included in the subarachnoid space) connected to ventricles. **(B)** Pre-processing of the MNI template (18). The bias of the template was first corrected using the N4 correction procedure (16). The brain, the cerebrospinal fluid (CSF), and the white matter (WM, including the major part of basal ganglia) masks were extracted using an iterative process based on Bayesian segmentation techniques (16, 17). Connected components of the CSF mask were then identified using ANTs image processing tools (http://stnava.github.io/ANTs/) to easily isolate the ventricles mask visually (not connected to the external CSF in this template). This mask was, then, dilated to keep potential incident lacunes connected to the ventricles of the follow-up (FU) MRI of the subject (kernel of 3 × 3 × 3 voxels). A distance map based on the Maurer distance (19) was also computed from the WM mask to keep only potential incident lacunes within this mask and exclude the most peripheral lesions (exclusion if d ≥ 0 mm). A basal ganglia mask was manually delineated by a neuroradiologist (RZ) and dilated to keep potential incident lacunes close to the inner edge of the WM mask (kernel of 3 × 3 × 3 voxels).

**Figure 2:**
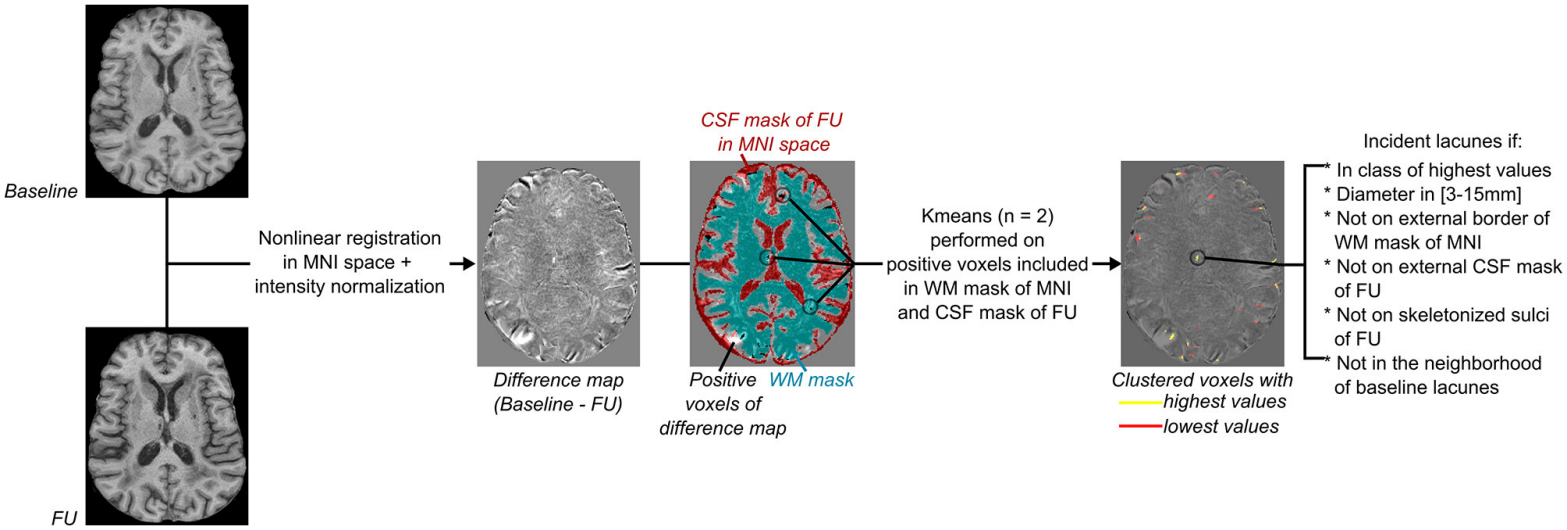
Flowchart of incident lacunes segmentation. From left to right: Both baseline and follow-up (FU) MRI were non-linearly registered in the MNI space (18, 20), normalized in intensity with FSL tools (https://fsl.fmrib.ox.ac.uk/fsl/fslwiki/), and then subtracted from each other to provide a difference map. As incident lacunes should appear with an MRI signal lower than non-lesioned cerebral matter, with an intensity close to the CSF signal of the FU MRI, only voxels of the difference map with a positive value and superimposed to the registered CSF mask of FU MRI were considered (refer to Figure 1A). Only clusters included in the cerebral white matter (WM) were kept, and their mask was defined on the MNI template (refer to Figure 1B). A K-means clustering with two classes was then performed on voxels considered likely lacunes and distinct from noise. The difference map was thresholded using the minimal value of the clustered class with the highest values. Only groups of voxels of diameter between 3 and 15 mm, not superimposed to the border of the WM MNI mask (refer to Figure 1B), to the external CSF mask (included in the subarachnoid space), or to the skeletonized sulci of FU MRI (refer to Figure 1A) were considered. Finally, clusters in the neighborhood of baseline lacunes were labeled separately from “incident” lacunes.

The lacunes at the baseline were first segmented semi-automatically as previously reported (14). The underlying pre-processing methods of MRI data are described in Figure 1. For each patient, the brain was extracted and non-linearly registered in the Montreal Neurological Institute (MNI) space (1 mm isotropic) with ANTs image processing tools (http://stnava.github.io/ANTs/) (18, 20), and then normalized in intensity with FSL tools (https://fsl.fmrib.ox.ac.uk/fsl/fslwiki/). The detection of incident lacunes (as defined according to the STRIVE criteria) was based on the MRI signal changes observed with the development of incident small cavity lesions containing CSF in the brain tissue (11, 12). Thus, the first follow-up data were subtracted from the baseline data for each patient. Since new lacunes should have lower T1 values than the corresponding area on baseline MRI, only voxels with positive values in the difference maps (baseline minus follow-up) were initially selected. Thereafter, only those located both in the MNI white matter (WM, including basal ganglia) and follow-up CSF masks were considered (17). Finally, a K-means clustering with two classes was performed to threshold the difference map obtained (21). The final threshold corresponded to the minimal value of the class with the highest intensities and allowed to select areas with the highest local MRI signal changes. Diameters of clusters were subsequently measured, and only those between 3 and 15 mm were selected to fulfill the STRIVE diagnostic criteria of lacunes (22). Finally, clusters superimposed to follow-up the external CSF (included in the subarachnoid space) and skeletonized sulci (15) or at the subcortical edges of the WM mask were automatically removed. It is noteworthy that clusters in the neighborhood of baseline lacunes were differentially labeled as “extension of former lacunes.” These different processing steps are summarized in Figure 2.

### 2.4. Data analysis

To evaluate the automatic segmentation, incident lesions were identified visually and rated independently by two experts in cSVD imaging research (HC, neurologist and RZ, neuroradiologist). The evaluation was performed with simultaneous visualization of the baseline and follow-up images already registered in the MNI space. After an inspection of all 3D-T1 MRI slices, each incident lacune was then marked, on a single MRI slice, on the screen by each rater using the Anatomist software (23) and blinded to the patient's status. Experts did not use any specific tool to measure the exact size of the lacune. They visually estimated their matching with the STRIVE criteria. A gold standard was thereafter obtained by consensus after a third reading in the presence of the two experts. For each incident lacune of the gold standard, the analysis of the performance of the algorithm to identify the same lesion was performed: If an incident lacune was automatically segmented in the neighborhood of the gold standard (gold standard dilated by a kernel of 3 × 3 × 3 voxels), it will be considered as “true positive”; if the algorithm missed this lacune, it will be considered as “false negative”; and if the algorithm segmented a lacune but not the gold standard, it will be considered as “false positive”. Then, the sensitivity and the F1-score between the proposed method and the gold standard were estimated for each patient with at least one incident lacune detected by the gold standard (lesion-level analysis). With all patients together, the number of incident lacunes provided by the automated and visual approaches was compared by the Wilcoxon signed-rank test (significance at 0.05). The presence of incident lacunes detected automatically and by the gold standard was finally binarized [yes (1) or no (0)] for each patient, for estimating the patient-level sensitivity, specificity, and F1-score of the detection obtained using the segmentation algorithm. All statistical comparisons and measures were calculated in Python3 (numpy version 1.21.5, scipy version 1.7.3).

## 3. Results

The median number of lacunes at the baseline was 7 (inter-quartile range IQR = [2; 11]). The average time required by each expert to visually detect the incident lacunes was approximately 8 min per MRI exam. Approximately 5 min per subject were required after this first step to define the gold standard by consensus. The algorithm took an average of 25 s per subject to fully segment incident lacunes and extended lacunes (on a Mac OS System, Big Sur; 3.8GHz Intel Core i7 8 cores; Memory: 16Go 2667 MHz DDR4; AMD Radeon Pro 5500 XT 8Go).

**Figure 3:**
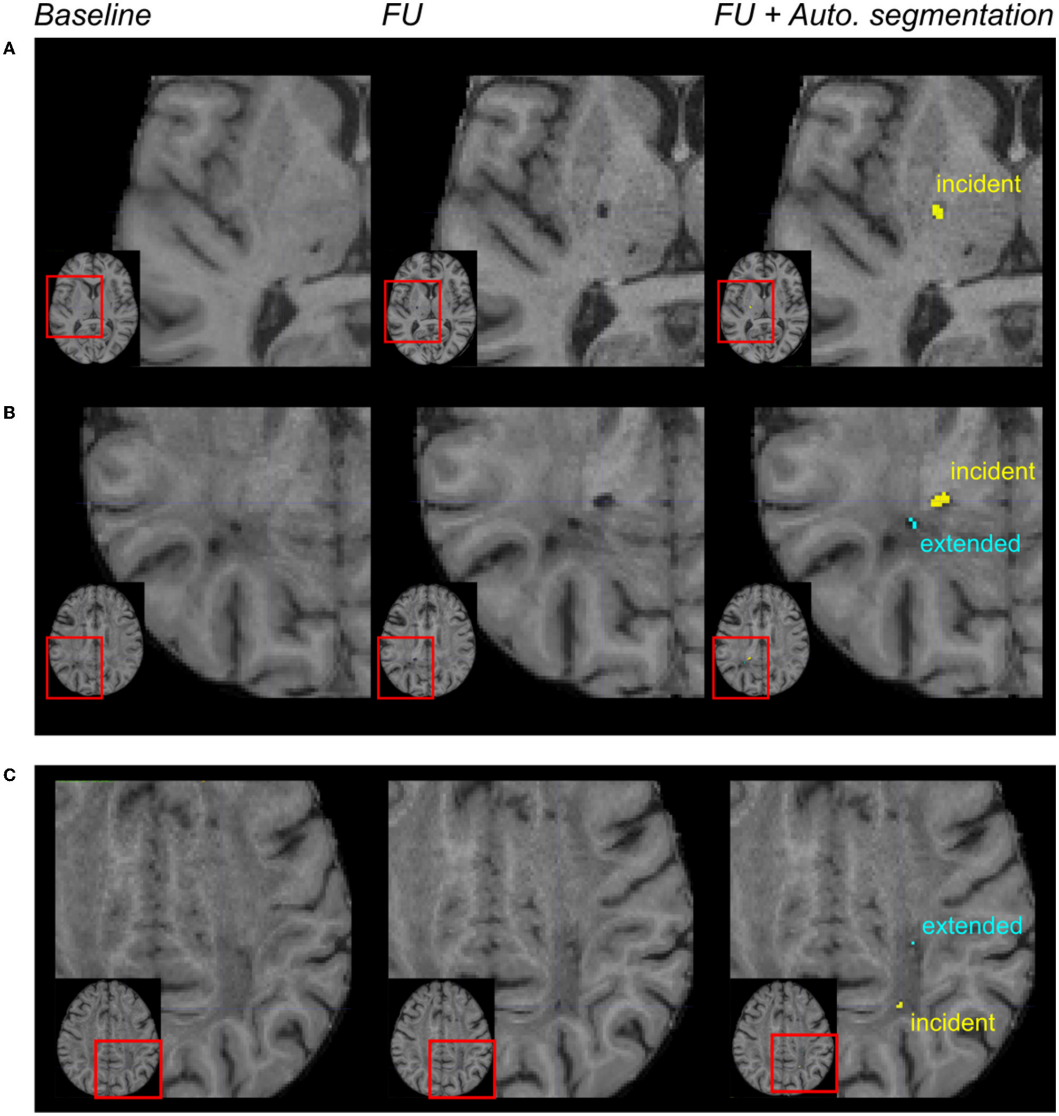
Automated incident lacunes segmentation and limitations. From left to right: magnification of MR images in different patients after the registration of the baseline and follow-up (FU) data in the MNI space. In the right column, the automated segmentation of lesions was superimposed on the FU MRI. In **(A, B)** incident lacunes were both identified by the experts and the algorithm. The automated method enabled to differentiate the incident lacune from the extension of a previous lacune **(B)**. In **(C)** these incident lacunes were only identified by the algorithm. The automated method enabled to differentiate the incident lacune from the extended part of a baseline lacune.

Among the 30 patients, the gold standard collected 45 incident lacunes with a median number per patient at 0.5 (IQR = [0; 2]), and the algorithm segmented 59 incident lacunes with a median number per patient at 2 (IQR = [0; 3]). With 20 lesions identified as “true positives,” 39 as “false positives”, and 25 as “false negatives,” the median sensitivity was 0.40 (IQR = [0; 0.67]), and the median F1-score was 0.50 (IQR = [0; 0.67]) (lesion-level analysis). With all patients together, both approaches provided a similar number of incident lesions (Wilcoxon test: *p* = 0.3). Based on the binarized presence of incident lacunes, the patient-level sensitivity, specificity, and F1-scores were equal to 0.80, 0.46, and 0.69, respectively. Figures 3A, B display incident lacunes, both detected by the experts and the algorithm and an “extended” part of a former lacune.

**Figure 4:**
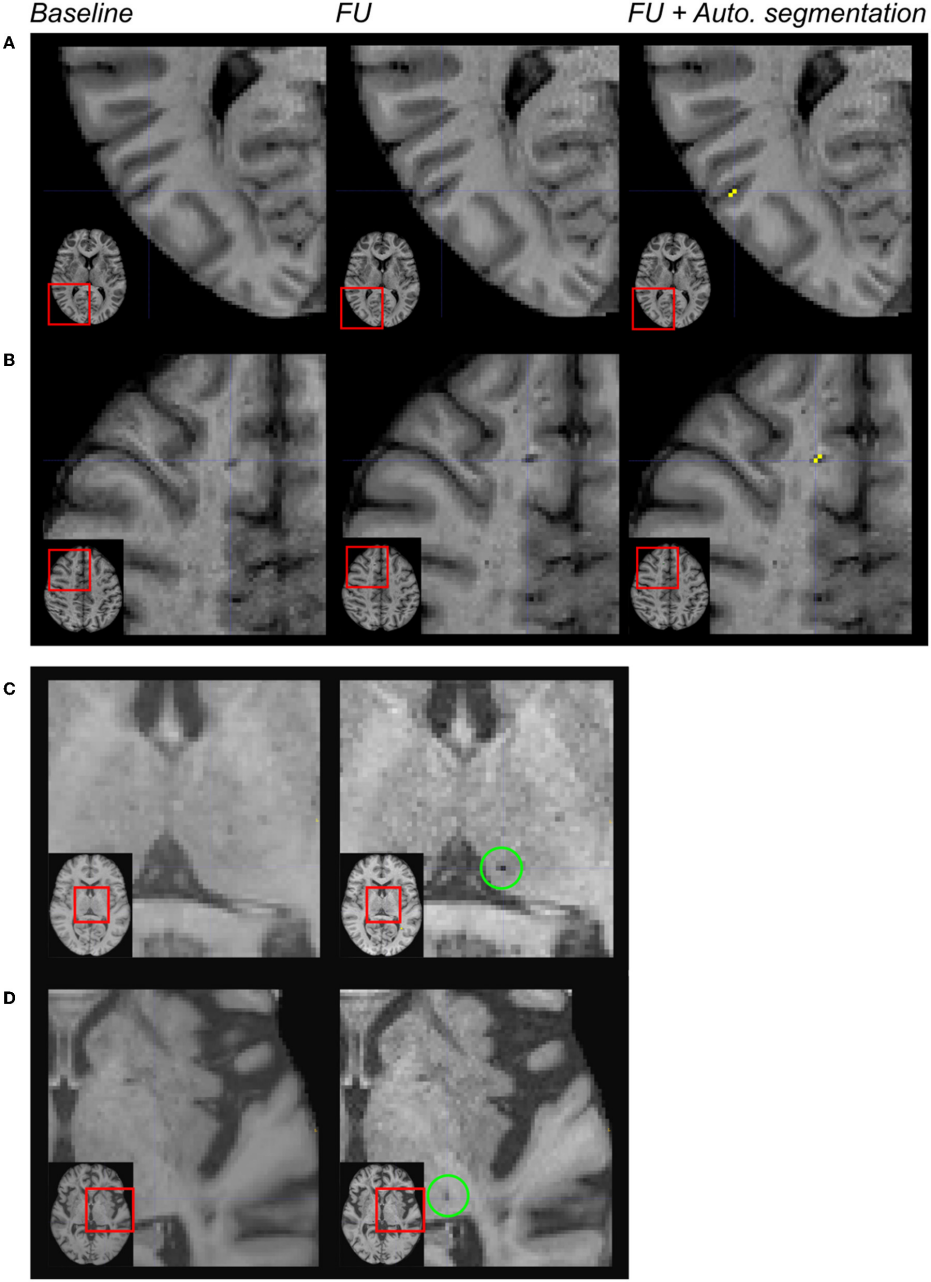
Limitations of the automated segmentation. From left to right: magnification of MR images in different patients after the registration of the baseline and follow-up (FU) data in the MNI space. In the right column, the automated segmentation of lesions was superimposed on the FU MRI. Because of brain atrophy in patients with CADASIL, false positive lacunes were identified by the algorithm after the enlargement of some cortical sulci **(A)**. Because the CSF signal has changed across acquisitions within perivascular spaces, false positive incident lacunes were also detected at such locations **(B)**. The algorithm failed because of the size of the cluster of interest (diameter < 3 mm) **(C)**. The algorithm failed because the region of interest did not belong to the CSF mask of follow-up data **(D)**.

A deep visual inspection of the results obtained using the algorithm showed that (1) most of the false positive lacunes were detected within the cortical sulci (18% of false positives) and/or in perivascular spaces, where some signal variations were detected during follow-up (36% of false positives); (2) some lacunes identified by the experts were missed by the algorithm because their diameter was < 3 mm (32% of false negatives) or because they were not included in the CSF mask obtained on follow-up MRI (24% of false negatives); and 3) there were also “true” lacunes detected by the algorithm but not by the experts, consequently unfairly identified as “false positive” (31% of the clusters so-called “false positives”) (refer to Figures 3, 4).

The manual removal by an expert of the most obvious false positives, mainly within the cortical sulci or perivascular spaces, was easy and fast (estimated time: 2 min per subject). The “true” lacunes detected by the algorithm but not by the experts were kept; the lacunes missed by the algorithm were not added. With these corrections, the number of “false positives” dropped to zero. For patients with at least one incident lacune truly detected (by the gold standard and/or the algorithm), the median sensitivity and the median F1-score to detect lacunes at lesion level reached 0.66 (IQR = [0.21; 1.00]) and 0.79 (IQR = [0.35; 1.0]), and the patient-level specificity and F1-score were 0.80 and 0.76, respectively.

## 4. Discussion

The results of this study showed first that it is possible to use conventional segmentation methods to automatically assess incident lacunes during the progression of cSVD, with a conventional computer system and with a fast processing time. This task is particularly challenging for different reasons: lacunes are small lesions and their diameter can be limited to 3 mm (13), and they can be confused with other small cavities of the brain containing CSF such as perivascular spaces (24) or with small signal changes related to brain movements or cerebral atrophy, particularly at the contours of the brain parenchyma (25, 26). Finally, the CSF signal itself may also vary with repeated MRI acquisitions and is not always consistent (27). Our approach allows, however, not only to measure the number of incident lacunes but also to estimate their location in the brain in further studies. These parameters might be of high interest to understanding the dynamics of structural tissue loss during the progression of cSVD.

The method proposed in this study appears highly sensitive at the group level. The main limitation of using only such an automated segmentation method is, however, the limited specificity and a significant risk of false positives of incident lesions at the individual level. Interestingly, the detailed analysis of errors observed using the algorithm showed that most difficulties encountered were not actually related to the segmentation method itself but rather to the very limits of defining an incident lacune operationally. Results showed that with the development of cerebral atrophy in cSVD, some voxels in the depth of the cortical sulci became apparent on follow-up MRI. Except for their location, their morphological features corresponded to the imaging definition of an incident lacune. In other cases, despite the use of refined methods to segment the brain and delineate its contours, the CSF signal was found to vary between two exams at the surface of the brain. Here, the corresponding voxels were also mistaken, as a cluster mimicking a small cavity developing in the brain. Additional studies are needed to improve brain contour delineation. On the contrary, we also found that some cavities, missed by the two experts, were appropriately recognized as lacunes by the algorithm. This illustrates some limits of the visual detection of incident lacunes in cSVD and the potential of such an automated approach. In line, very small incident lacunes were sometimes identified visually by the experts but not by the algorithm. Here again, the error was not related to the automated method but to the definition of the lacune itself, imposing a diameter of at least 3 mm, which no examiner could achieve from only visual examination. Preliminary tests (not shown) based on a shape constraint to only keep round lesions for lacunes showed an obvious underestimation of lesions because some of them were ovoid or different in shape. Further investigations led to include a shape constraint, mainly for removing elongated lesions that did not correspond to lacunes.

No obvious effect related to the different MR protocols in use was detected. Additional studies are, however, needed to determine whether our algorithm is actually insensitive to various MR acquisitions.

Most of the errors observed using the automated method were detected at the cortical surface or in perivascular spaces. They are easy to recognize visually because of their topographic distribution. Thus, we tested whether a combined approach using an automatic segmentation followed by a manual correction of these predictable errors was feasible and could be obtained easily. The results showed that such false positive lesions could be identified and removed quickly through manual correction of voxels identified by the algorithm. Moreover, the combined task (automated algorithm + manual correction) was found to last on average 2.25 min per case while 8 min were needed to detect and identify incident lacunes by the experts. Finally, using this combined approach, the median sensitivity for detecting individual lesions on MRI increased up to 0.66, a value that can be considered satisfactory at the case level. A sensitivity and specificity of 0.80 were even obtained at the group level. Therefore, we believe that such a combined approach can already be used to separate patients very quickly from those without incident lacunes in large MRI datasets and to quantify the number of incident lacunes at the individual level, even in the presence of a severe cSVD.

The strengths of this study are multiple. The search for incidental lacunes was carried out in a group of patients with particularly severe small vessel disease and who presented multiple lesions at the baseline, thus under particularly demanding conditions. The lesion segmentation was based on readily available tools, and the treatment pipeline can be easily replicated. The results are immediately applicable and were obtained from clinical MRI data. Some limitations are also evident. External validation in a very large cohort of patients with mild to severe forms of cSVD would be useful to consolidate these findings. Some technical developments for removing the voxels identified at the surface of the cerebral cortex have to be investigated and should further improve this automated segmentation method. Finally, this approach does not exclude others such as the development of the identification of lacunes by supervised learning methods [for example, based on the convolutional neural network (28, 29)], which seem to offer other advantages such as better accuracy of the detected lesions but also disadvantages such as more important computing resources (days to constitute the training set and dozens of hours to perform the segmentation with an adapted computing system including large memory capacity). Different approaches have to be compared in terms of accuracy and also computation facility.

In conclusion, we have developed an automatic segmentation approach to assess incident lacunes in cSVD and showed that by combining some quick manual correction of systematic errors, such an approach can be already proposed to segment incident lacunes in large databases of cSVD. In the future, additional technical developments are warranted to further improve this promising approach, which is already capable of overcoming some unexpected limitations of visual assessment. These efforts will also be needed to further increase the sensitivity and specificity of an automated method without any human intervention.

## Data availability statement

The raw data supporting the conclusions of this article will be made available by the authors, without undue reservation.

## Ethics statement

The studies involving human participants were reviewed and approved by the Independent Ethics Committee (updated agreement CEEI-IRB-17/388) and was conducted in accordance with the Declaration of Helsinki and guidelines for Good Clinical Practice and General Data Protection Regulation (GDPR) in Europa. The patients/participants provided their written informed consent to participate in this study.

## Author contributions

HC designed the project. JL, RZ, and HC designed the experiments. JL developed the method and analyzed and interpreted the data. RZ, LG, and HC provided the reference segmentation for the baseline and/or follow-up. JG and EJ managed the image acquisition. FF managed the project. JL and HC drafted the manuscript. RZ and EJ revised the manuscript. All authors have read and approved the submitted version of the manuscript.
